# Thoracic Endometriosis: A Presentation of an Uncommon Disease in a Black African Woman

**DOI:** 10.1155/2022/2380700

**Published:** 2022-03-17

**Authors:** John Omotola Ogunkoya, Taiwo Olufemi Solaja, Akinwale Folarin Ogunlade, Marion Itohan Ogunmola

**Affiliations:** ^1^Division of Respiratory Medicine and Allergy, Babcock University Teaching Hospital, Ilishan Remo, Ogun State, Nigeria; ^2^Benjamin Carson Senior College of Health and Medical Sciences, Babcock University, Ilishan Remo, Ogun State, Nigeria; ^3^Histopathology Department, Babcock University Teaching Hospital, Ilishan Remo, Ogun State, Nigeria

## Abstract

**Introduction:**

Endometriosis is defined as a chronic gynecologic disease which is characterized by the presence of endometrial glands and stroma in anatomical sites and organs outside the uterine cavity. The exact prevalence of endometriosis is difficult to determine because many women remain asymptomatic. However, endometriosis affects about 10% to 15% of women. Thoracic endometriosis (TES) is the most common endometriosis outside the abdominopelvic cavity. It refers to endometriosis within the thoracic cavity including the lung parenchyma, diaphragm, and pleural surfaces. It can manifest as catamenial chest pain, pneumothorax, hemoptysis, hemothorax, catamenial haemoptysis, and pulmonary nodules. *Case Summary*. A 39-years-old married female presented with recurrent right-sided chest pain of 22 years duration, recurrent cough of more than 20 years and progressive breathlessness of a month duration. The chest pain is pleuritic, and it often starts few days to the onset of her menses and lasts throughout menstrual flow only to abate after the stoppage of menstrual bleeding. Cough was unproductive, paroxysmal often worse with worsening chest pain. It disappears after the end of menstrual bleed. Breathlessness was initially on mild to moderate exertion before progressing to occasional breathlessness at rest. No history of orthopnea, paroxysmal nocturnal dyspnea, and pedal swelling was found. Over the years, she had presented to several clinics where she was said to have menstrual pain referred to the chest.

**Conclusion:**

Diagnosis of extrapelvic endometriosis can be challenging and delayed because it presents in a myriad of ways and in some cases, it may be difficult to link symptoms and the menstrual cycle.

## 1. Introduction

Endometriosis is defined as a chronic gynecologic disease which is characterized by the presence of endometrial glands and stroma in anatomical sites and organs outside the uterine cavity [1]. Endometriotic deposits can be found almost anywhere; the commonly involved sites being the ovaries, the posterior broad ligament, the anterior cul-de-sac, the posterior cul-de-sac, and the uterosacral ligament in that order [1–4]. It can occur less commonly in remote locations such as the intestine, thoracic cavity, and other organs [3]. It is an estrogen-dependent disease that manifests as subfertility, chronic pelvic pain, fatigue, dysmenorrhea, dyspareunia, dysuria, and dyschesia [1, 3, 5]. The exact prevalence of endometriosis is difficult to determine because many women remain asymptomatic [1]. However, endometriosis affects about 10% to 15% of women [6–8]. In symptomatic women, prevalence rate can be as high as 50% or more [7, 8].

Various hypothesis has been put forward to explain endometriosis: Sampson's theory, coelomic metastatic theory, müllerian remnant theory, lymphatic and vascular metastasis theory, and the stem cell theory among others [1, 9]. Sampson's theory, one of the oldest theories on endometriosis, proposed that the retrograde flow of endometrial cells via the fallopian tubes into the pelvic cavity during menstruation was responsible for endometriosis [7, 10]. Another theory, the coelomic metaplasia theory, postulates that metaplasia of specialized cells, present in the mesothelial lining of the peritoneum under the influence of cytokines and growth factors of the endometrial stroma, was the cause of endometriosis [11, 12]. Endometriosis is also thought to originate from müllerian remnant that fails to migrate or differentiate properly during fetal development (which is the müllerian remnant theory) or from circulating blood cells that differentiate into endometriotic deposits [13, 14]. The lymphatic and vascular metastasis theory proposes that endometrial tissue can through the lymphatics and blood vessels get transferred to remote areas like the brain or the pleura [15, 16]. Each of these theories cannot adequately explain the pathogenesis of endometriosis especially when found outside the abdomen and pelvis [1]. It therefore follows that endometriosis is a complex disease involving the interactions of hormonal, genetic, immune, and environmental factors [1, 17].

## 2. Case Summary

A 39-years-old married female presented via the emergency department of the hospital on account of recurrent right-sided chest pain of 22 years duration, recurrent cough of more than 20 years, and progressive breathlessness of a month duration. The chest pain started in her teenage years, sharp, pleuritic sometimes aching in nature with a score of 5–6 on a scale of 1–10 and not severe enough to negatively impact her day-to-day activities. It often starts a few days to the onset of her menses, lasts throughout menstrual flow only to abate after the stoppage of menstrual bleeding. Cough was unproductive, paroxysmal often worse with worsening chest pain. It disappears after the end of menstrual bleeds. No associated hemoptysis, weight loss, or drenching night sweats were found. No history of fever and no change in appetite or other gastrointestinal symptoms was found.

Breathlessness started a month before presentation, initially on mild to moderate exertion before progressing to occasional breathlessness at rest. It was first noticed while climbing a flight of stairs but now even while bathing or putting on her dress. No history of orthopnea, paroxysmal nocturnal dyspnea, and pedal swelling was determined.

Over the years, she had presented to several clinics where she had complained of crampy chest pain which usually started during her menstrual flow. She was, however, referred to the emergency department of this hospital by her GP when the breathlessness worsened. She has no known allergy and is not on any routine medication. Patient does not drink alcohol nor smokes cigarettes and had not had any exposure to second hand smoke. She is married in a monogamous setting with 2 children. She was diagnosed and treated for acid peptic disease 3 years prior to presentation. Her genotype gotten via haemoglobin electrophoresis is AA.

At presentation, she was in mild respiratory distress, anxious, not pale, afebrile to touch, mildly dehydrated, not cyanosed, no palpable peripheral lymphadenopathy, and no pedal edema. Her respiratory rate was 38 cycles/min, oxygen saturation was 93% in room air with normal arterial blood gas result (pH = 7.38, PaO_2_ = 88 mmHg, PaCO_2_ = 42 mmHg, and HCO_3_ = 24 mEq/L), trachea was central. Chest examination was suggestive of right middle and lower lobes consolidation with right-sided effusion. Cardiovascular examination was essentially normal. The abdomen was full, moved with respiration. No area of tenderness and no palpable organ enlargement were found.

**Figure 1:**
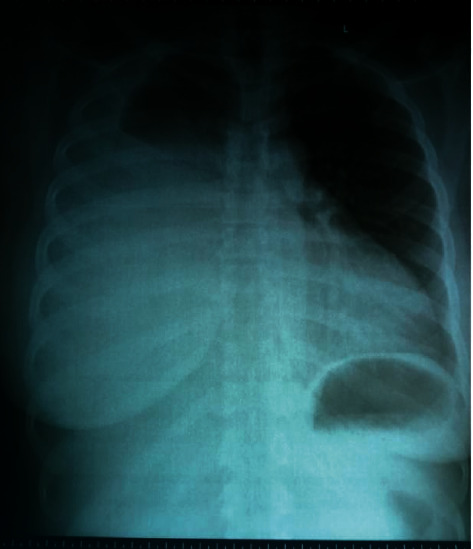
A plain chest radiograph of the study subject in anterioposterior view showing massive right-sided pleural effusion.

Initial assessment was a right-sided pleural effusion secondary to pulmonary tuberculosis, to keep in view disseminated tuberculosis. Samples were taken for full blood count, electrolytes, urea and creatinine, hepatis B surface antigen, hepatitis C antibody, human immunodeficiency virus (HIV) screening, liver function test, plain chest radiograph, and chest computerized tomographic scan. She was commenced on intravenous paracetamol 300 mg prn, intravenous 5% dextrose saline 500 mls 6 hourly and oxygen via nasal prong to raise oxygen saturation above 96%. Chest radiograph (PA view) showed massive right-sided pleural effusion (Figure 1). Consult was then sent to cardiothoracic unit for closed thoracotomy tube drainage (CTTD).

**Figure 2:**
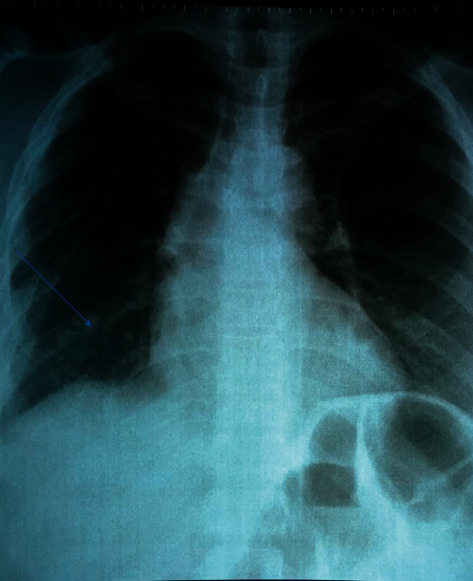
A plain chest radiograph of the study subject in anterioposterior view showing a near total re-expansion of the lung after chest tube drainage.

On the second day of admission, patient was seen by CTSU team and counseled on the need for closed tube thoracotomy. This was conducted, and the chest tube drained about 2600 mls of bloody fluid in the first 24 hours. The patient was provided with incentive spirometer, and sample of the pleural fluid was sent for GeneXpert, cytology, immunohistochemistry, lactate dehydrogenase assay (pleural fluid and serum), microscopy, culture, and sensitivity. The cardiothoracic team (CTSU) made a presumptive diagnosis of catamenial hemothorax because of positive history of cyclical right-sided chest pain during menstrual flow, and a review by the gynecologist was requested. The gynecologist reviewed and was in agreement with the CTSU. The post chest tube check X-ray showed significant re-expansion of the lung after 24 hours (Figure 2).

**Figure 3:**
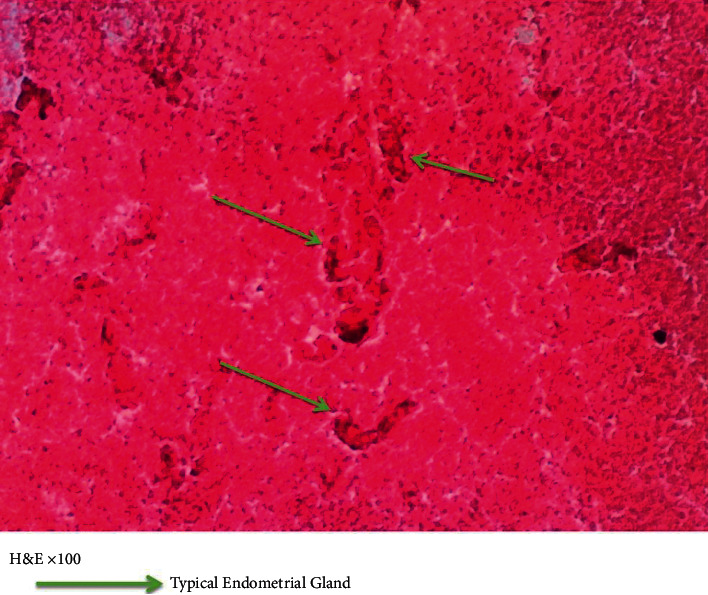
Hematoxylin and eosin staining of pleural fluid sample showing the presence of typical endometrial glands.

**Figure 4:**
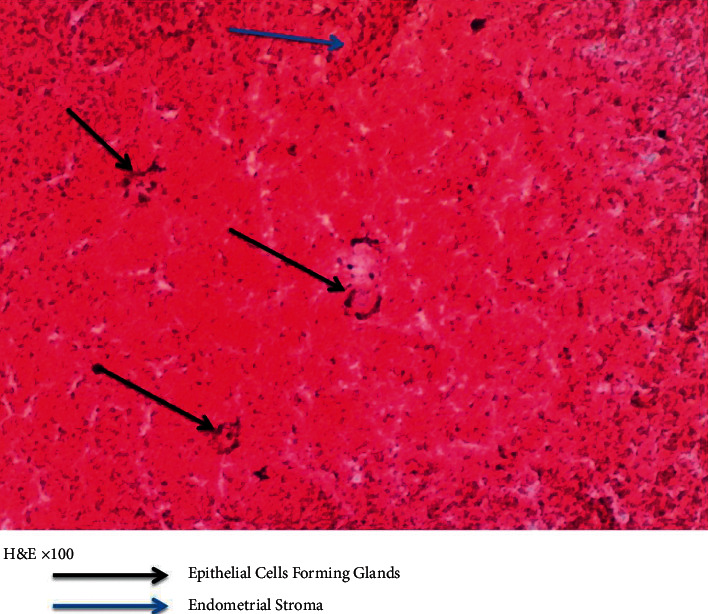
Hematoxylin and eosin staining of pleural fluid sample showing the presence of endometrial stroma and epithelial cells forming glands.

Other lab results were as follows:

(1) Pleural fluid GeneXpert result: *Mycobacteria tuberculosis* was not detected in the pleural fluid.
(2) Pleural fluid cytology revealed mainly amorphous eosinophilic material with few degenerating cells.
(3) Pleural fluid m/c/s yielded no growth.
(4) LDH
  - Serum-221 mg/dl
  - Pleural-229 mg/dl
  - Ratio-1.03
(5) Total protein:
  - Pleural fluid-224 mg/dl (<300 mg/dl)
  - Serum protein-7.1 mg/dl
(6) Human immunodeficiency virus screening was negative
(7) Pleural fluid cytology report (Figures 3 and 4) demonstrated the presence of typical endometrial glands, epithelial forming glands, and endometrial stroma.

**Figure 5:**
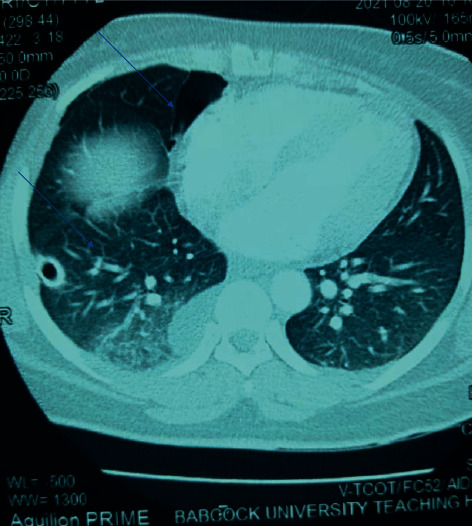
Chest CT scan of the chest of the patient showing a near-complete expansion of right lung (90–95%) with mild right-sided residual effusion and no obvious parenchymal lesion seen.

On the 3^rd^ day of admission, the chest tube kept draining the serosanguinous fluid. The chest CT scan result showed a near-complete expansion of right lung (90–95%) with mild right-sided residual effusion. No obvious parenchymal lesion was seen (Figure 5). About 8 days after admission, chest drain had become inactive and patient commenced chest physiotherapy. A repeat chest X-ray showed residual right-sided pleural effusion which was mild. Chest tube was subsequently removed on the 10^th^ day of admission following a 24-hour period of inactivity and patient was discharged on the 11^th^ day of admission. She was given appointment to see the pulmonologist in the chest clinic 2 weeks after discharge. She came for follow-up as scheduled with no symptom and no clinical evidence of recollection of fluid in the pleural cavity. She was informed of possible need for segmentectomy or lobectomy with sample sent for histology if symptoms worsens or if onset of recurrent hemoptysis. She was subsequently referred to the gynecologist for follow-up.

## 3. Discussion

The diagnosis of thoracic endometriosis (TES) is a diagnosis of exclusion with atypical clinical history. However, the relationship of the onset and evolution of symptoms and menstrual cycle is a classic characteristic feature. The diagnosis of TES includes careful evaluation of the patient's history, physical examination supported by laboratory tests such as chest radiograph and CT scan and more importantly the review of the cytology of pleural fluid sample. Biopsy has no major role in the diagnosis of TES but postoperative histopathological assessment can help strengthen diagnosis [18].

Thoracic endometriosis (TES) is the most common endometriosis outside the abdominopelvic cavity [19]. It refers to endometriosis within the thoracic cavity including the lung parenchyma, the diaphragm and pleural surfaces [20]. It can manifest as catamenial chest pain, pneumothorax, and hemoptysis, hemothorax, catamenial hemoptysis, and pulmonary nodules [20, 21]. The mean age at presentation of TES was 35 years with a range from 15 years to 54 years [22]. It most commonly presents with pneumothorax followed by hemothorax and hemoptysis which were not seen in this index patient. The right hemithorax is involved in more than 90% of all manifestations except for pulmonary nodules [22]. Approximately 50–84% of women with pelvic endometriosis also have thoracic endometriosis [22].

Diagnosis of extrapelvic endometriosis can be challenging and delayed because it presents in a myriad of ways and in some cases, it may be difficult to link symptoms and the menstrual cycle. The interval between the onset of symptoms and the establishment of a definitive diagnosis of thoracic endometriosis ranges between 8 and 16 months [23].

For women with severe symptoms and recurrence of TES surgical relief vis a vis segmentectomy or lobectomy is the treatment of choice [24]. However, medical therapies using oral or parenteral progestogen contraceptives, gonadotropin-releasing hormone agonist, nonsteroidal anti-inflammatory drugs, and androgens are very useful in relieving symptoms [25].

## Data Availability

No data sets were used other than the medical record of the patient.
